# Increasing Access to Mental Health Care With Breathe, an Internet-Based Program for Anxious Adolescents: Study Protocol for a Pilot Randomized Controlled Trial

**DOI:** 10.2196/resprot.4428

**Published:** 2016-01-29

**Authors:** Amanda S Newton, Lori Wozney, Alexa Bagnell, Eleanor Fitzpatrick, Sarah Curtis, Mona Jabbour, David Johnson, Rhonda J Rosychuk, Michael Young, Arto Ohinmaa, Anthony Joyce, Patrick McGrath

**Affiliations:** ^1^University of AlbertaEdmonton, ABCanada; ^2^IWK Health CentreHalifax, NSCanada; ^3^Dalhousie UniversityHalifax, NSCanada; ^4^University of OttawaOttawa, ONCanada; ^5^University of CalgaryCalgary, ABCanada

**Keywords:** anxiety, etherapy, cognitive behavioral therapy, adolescents, mental health, Internet, intervention, pilot, randomized controlled trial

## Abstract

**Background:**

There is a demand to make first-line treatments, including cognitive behavioural therapy (CBT) for adolescent anxiety disorders, more widely available. Internet-based CBT is proposed to circumvent access and availability barriers and reduce health care system costs. Recent reviews suggest more evidence is needed to establish the treatment effects of Internet-based CBT in children and adolescents and to determine related economic impacts.

**Objective:**

This pilot trial aims to collect the necessary data to inform the planning of a full-scale RCT to test the effectiveness of the Internet-based CBT program Breathe (Being Real, Easing Anxiety: Tools Helping Electronically).

**Methods:**

We are conducting a 27-month, 2-arm parallel-group, pilot randomized controlled trial (RCT). Outcomes will inform the planning of a full-scale RCT aimed to test the effectiveness of Internet-based CBT with a population of adolescents with moderate to mild anxiety problems. In the pilot RCT we will: (1) define a minimal clinically important difference (MCID) for the primary outcome measure (total anxiety score using the Multidimensional Anxiety Scale for Children); (2) determine a sample size for the full-scale RCT; (3) estimate recruitment and retention rates; (4) measure intervention acceptability to inform critical intervention changes; (5) determine the use of co-interventions; and (6) conduct a cost-consequence analysis to inform a cost-effectiveness analysis in the full-scale RCT. Adolescents aged 13-17 years seeking care for an anxiety complaint from a participating emergency department, mobile or school-based crisis team, or primary care clinic are being screened for interest and eligibility. Enrolled adolescents are being randomly allocated to either 8 weeks of Internet-based CBT with limited telephone and e-mail support, or a control group with access to a static webpage listing anxiety resources. Adolescents are randomly assigned using a computer generated allocation sequence. Data are being collected at baseline, treatment completion, and at a 3-month follow-up.

**Results:**

Currently, adolescents are being enrolled in the study. Enrolment is taking place between March 2014 and February 2016; data collection will conclude May 2016. We expect that analysis and results will be available by August 2016.

**Conclusions:**

In many communities, the resources available for front-line anxiety treatment are outweighed by the need for care. This pilot RCT is an essential step to designing a robust RCT to evaluate the effectiveness of an Internet-based CBT program for adolescents with moderate to mild anxiety problems.

**Trial Registration:**

Clinicaltrials.gov NCT02059226; http://clinicaltrials.gov/ct2/show/NCT02059226 (Archived by WebCite at http://www.webcitation.org/6epF8v7k4)

## Introduction

Anxiety disorders are the most common mental disorders diagnosed in adolescence, with prevalence before age 18 reported as greater than 30% [1,2]. The median age of onset is 11 years, making these disorders some of the earliest to develop [3]. Early onset disorders often follow a chronic course [4] and can significantly interfere with interpersonal relationships, academic performance and daily functioning [5-7]. Anxiety disorders range in severity and specific symptomology, but the more frequently diagnosed disorders in adolescents include social anxiety disorder (social phobia) [8-10], generalized anxiety disorder [8,11,12], and specific phobia [8,12]. The most prevalent is social anxiety disorder, with studies showing a two-fold increase in the prevalence of this disorder during teenage years [13,14]. The high prevalence of social anxiety disorder in adolescence can be understood in the context of developmental and environmental transitions (eg, puberty, dating, new schools, peer experiences) [9]. Undetected or undertreated, these disorders present significant future risk for adult anxiety disorders, educational underachievement, suicidality, depression, substance abuse and future hospitalization [5-7,15-19].

Cognitive behavioral therapy (CBT) as a first-line treatment for anxiety disorders in children and adolescents is well established [20,21,22] and supported by meta-analytic work [23,24,25]. CBT combines systematic exposure to feared situations with skills training and the learning of activities to help replace anxious thoughts about feared situations with more adaptive thoughts [20,21]. These treatment elements are based on the premise that repeated exposure to feared situations results in desensitization, reducing anxiety and avoidant behaviors, and improving functioning. Relaxation strategies, such as imagery and deep breathing, are included in skills training to provide ways to manage discomfort during repeated exposures [21].

Trained mental health professionals have traditionally delivered CBT, but the structured and sequential nature of CBT translates well to computer-based delivery. Within this approach, the use of the Internet as a delivery mode provides researchers with unobtrusive ways to capture data and examine access patterns, compliance, and usability issues [26]. Internet-based delivery also circumvents multiple barriers to receiving in-person CBT including: social stigma, direct and incidental costs (eg, time out of school), lack of trained deliverers, and inconvenient service times and locations [27]. This delivery method may also increase the number of young people seeking professional help. A published paper in 2002 reported that over a one-year timeframe, only 8.3% of young people with recent onset anxiety and 21.4% with chronic anxiety had sought professional help [28]. While Internet-based CBT is considered lower-intensity than in-person treatment, the majority of adolescents with anxiety disorders experience mild to moderate distress and impairment, making treatment availability paramount. Thus, Internet-based CBT is proposed to reduce demand for therapy delivered in-person, introduce cost savings, and increase timely access to recommended treatment [29].

Three recent systematic reviews of the literature have shown that self-led, computer-based and computer-assisted CBT, in combination with in-person or therapist-guided care, are viable and effective treatment approaches for children and adolescents [30,31,32]. Studies of treating adolescents using Internet-based CBT are limited to two [33,34], but provide important evidence and methodological considerations for other treatment studies. In a study targeting social fears (mainly centered on public speaking) in high school students who met the diagnostic criteria for social anxiety disorders, Tillfors et al. found that significant reductions in anxiety symptoms and improvements to mood were achieved post-intervention and maintained at one-year follow-up [34]. These treatment effects were noted as comparable to those achieved through traditional face-to-face CBT. Focusing on the treatment of adolescents who had a “markedly disturbing/disabling” anxiety disorder (predominantly generalized anxiety disorder and social phobia), Spence et al. also found that Internet-based CBT with minimal therapist support was equally efficacious as face-to-face CBT delivery in reducing anxiety symptoms post-intervention and up to one-year post-treatment [33]. The parents of participating adolescents also took part in this online program. In both studies, methods to improve treatment compliance, and the impact of compliance on treatment outcome, were noted as issues that required further investigation. Tillfors et al. reported that, of nine online modules available to complete within a 9-week period, the average number of completed modules was 2.9 [34]. Spence et al. noted that on average, adolescents and parents completed 7.5/10 and 4.48/5 sessions, respectively [33].

While the reported treatment effects of Internet-based CBT for treating adolescent anxiety are promising [33,34], published systematic reviews highlight important methodological issues that future studies must consider, in order to mature the evidence base [30,31,32]. Two reviews reported that the overall quality of the outcomes reported by studies of computerized CBT, including Internet-based CBT for adolescents, was *low* to *very low* [30,32]. Key limitations of the evidence base that were identified included: small sample sizes, intervention and comparator (waitlist or active control) differences between studies, and inconclusive treatment effects when anxiety was self-rated (versus clinician rated) [30,31,32]. Many of these limitations were also noted in the Tillfors and Spence studies [33,34]. Reviews to date have recommended that higher quality evidence is needed to firmly establish the treatment effects of computerized CBT, and that the study of associated economic impacts is needed [30,31,32].

We designed a pilot randomized controlled trial (RCT) that addresses several of the limitations highlighted in the literature to date. This pilot trial aims to collect the necessary data to inform the planning of a full-scale RCT to test the effectiveness of the Internet-based CBT program *Breathe* (Being Real, Easing Anxiety: Tools Helping Electronically). In the pilot RCT we will (1) define a minimal clinically important difference (MCID), as defined by adolescents, for the primary outcome measure, (2) determine a sample size for a full-scale RCT, (3) estimate recruitment and retention rates to determine the number of study sites needed, (4) measure intervention acceptability to inform critical intervention changes, (5) determine the use of co-interventions, and (6) conduct a cost-consequence analysis to inform a cost-effectiveness analysis for a full-scale RCT.

## Methods

### Study Design

**Figure 1:**
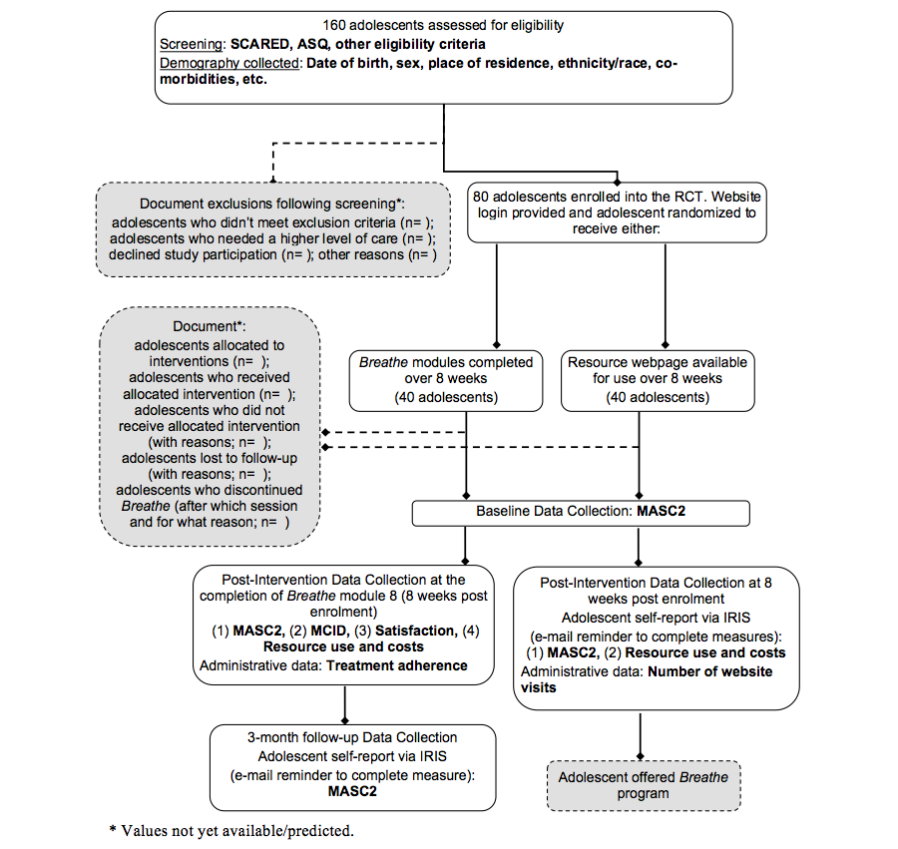
Trial flow diagram.

We are conducting a 27-month, 2-arm parallel-group, pilot RCT. Eighty adolescents will be randomly assigned to either 8 weeks of Internet-based CBT with telephone and e-mail support (the *Breathe* program), or a control (minimal intervention) group with 8 weeks of access to a static webpage with general anxiety resources and information. Study enrolment is taking place from March 2014 to February 2016; data collection will conclude May 2016. Details of the study design are illustrated in Figure 1. The study has been approved by host institutions’ Research Ethics Boards.

### Participants

Participants are adolescents from three Canadian provinces (Alberta, Ontario, and Nova Scotia) seeking care from a pediatric emergency department, mobile or school-based mental health team, or primary care setting that is participating as a study recruitment site. Study inclusion criteria are: (1) ages 13 to 17 years, (2) self-reported mild to moderate anxiety, (3) ability to read and write English, (4) regular access to a telephone and a computer system with high speed Internet service, and (5) ability to use a computer to interact with web material. Adolescents aged 13 to 14 years must also have a consenting parent.

Adolescents are screened for study inclusion criteria via a secure online recruitment process or telephone-based recruitment phone call. Demography is collected during the screening process to characterize adolescents who meet and do not meet clinical eligibility. Screening criteria (1), (3), (4), and (5) are assessed through yes/no answers. We are using the Screen for Child Anxiety Related Emotional Disorders (SCARED) [35] to screen for mild-to-moderate anxiety symptoms (eligibility criteria #2). The SCARED is a 41-item self-report screen for symptoms of panic disorder, social phobia and anxiety disorder, and general anxiety disorder in clinical and community adolescent samples, based on DSM-IV criteria [36,37,38]. Adolescents can report any experience of anxiety (ie, responds to any of the 41 items of anxiety symptoms/experiences as being “Somewhat/Sometimes True” or “Very/Often True”) to be considered eligible for study participation.

We are also screening for risk of suicide using the Ask Suicide-Screening Questions (ASQ) [39]. The ASQ is a brief 4-item instrument originally validated for use in emergency departments with high sensitivity for identifying the risk of suicide [39]. A “yes” response to questions 1 (“In the past few weeks, have you wished you were dead?”), 2 (“In the past few weeks, have you felt that you or your family would be better off if you were dead?”) or 4 (“Have you ever tried to kill yourself?”) on the ASQ involves a safety-follow up phone call from a research team member to evaluate intent/severity/immediacy of risk before deciding on adolescent eligibility. Adolescents who respond “yes” to question 3 on the ASQ (“In the past week, have you been having thoughts about killing yourself?”) are excluded from the study and receive brief telephone based support from a research team member to evaluate intent/severity/immediacy of risk and encourage the adolescent to seek further mental health care/crisis care.

### Procedure and Randomization

Potentially eligible participants (and their parents) can receive oral, written, and/or online information about the study. In the Canadian provinces involved in this study, there is no specified age of consent. A minor’s ability to consent or assent is decided after assessing capacity to consent. In this study, 15 to 17 year-olds will be allowed to consent to the study on their own behalf. We will require parental consent for adolescents aged 13 to 14 years. This decision was not based on any age of consent law; we want to acknowledge the role that parents have in their younger adolescent’s activities and their ability to provide perspective on their child’s potential participation in the study. Requiring parental consent will provide us with a formal mechanism to engage with parents before study enrolment. We will seek assent from adolescents aged 13 to 14 years even if they were assessed as being able to consent.

Randomization takes place after informed consent/assent is obtained. Adolescents are randomly assigned using a computer-generated allocation sequence. Participants are randomly allocated to one of two treatment conditions and receive this allocation information via e-mail from a graduate student trainee affiliated with the project. The e-mail also provides login/website information to the allocated intervention. Given the study objectives, the pilot was designed as open-label.

### Measures

#### Primary Outcome Measure

Change in anxiety symptoms in adolescents will be measured using the total score from the Multidimensional Anxiety Scale for Children (MASC2; 50 items). The MASC2 is one of the most widely used self-report measures in clinical trials in adolescents with anxiety disorders. It assesses physical symptoms, social anxiety, harm avoidance, separation/panic, and total anxiety, and has excellent 3-month test-retest reliability [40] and excellent validity [41,42]. Adolescent self-report is desirable because of the internalizing nature of anxiety [43]. The MASC2 is administered online via Intelligent Research Intervention Software (IRIS) to adolescents in the treatment group at three data collection time points: baseline (pre-treatment), 8 weeks/end of 8^th^ *Breathe* module (post-treatment) and 3 months post-treatment (follow-up). Adolescents in the control group complete the MASC2 at baseline and 8 weeks after study enrolment via IRIS. Table 1 outlines screening and outcome measurement time points.

**Table 1:** Outcome measurement schedule.

	Study time points					
	Study eligibility screening	Baseline	8 weeks (postintervention)	20 weeks (3-month follow-up)	End of study enrolment period (22 months from trial start)	End of data collection period (27 months from trial start)
Demography	X					
SCARED	X					
ASQ	X					
Co-intervention use/costs			X			
MASC2		X	X	X		
MCID			X			
Treatment satisfaction			X			
Treatment adherence			X			
Number of adolescents screened					X	
Number of adolescents enrolled					X	
Number of adolescents retained at 3-month follow-up						X

#### Secondary Outcome Measures

All secondary outcome data are collected via self-report using IRIS. The MCID will be determined for the primary outcome measure following module 8 completion (post-intervention). Adolescents allocated to the treatment condition are asked to indicate the minimum change in anxiety for which they would consider treatment participation. We will use the MCID to guide interpretation and application of outcome change in a full-scale RCT. To estimate the MCID, we will use adolescents’ global ratings of change on a 10-point Likert scale (-5 to +5), a commonly used anchor [44,45].

Satisfaction will be measured in adolescents allocated to the treatment condition at the end of module 8 (post-intervention) to infer intervention acceptability. A 12-item instrument developed by the research team will measure satisfaction based on the adolescent’s report of the program’s engagement and sense of safety/privacy, expectations and usefulness, communication, and technical (intervention) management. For 10 statements, a 5-point Likert response format ranging from strongly agree (score 5) to strongly disagree (score 1) will be used. Scores range from 10 to 50 with scores of 40 or greater indicating higher acceptability. Two open-ended questions to inform the larger trial will allow adolescents to identify challenges or barriers that were faced in taking part in the trial, and the extent to which adolescents used the skills learned.

Treatment adherence will be measured at 8 weeks to further evaluate intervention acceptability. Adherence is measured by documenting the number of treatment modules and homework tasks completed. We will also record the number of tailored modules completed by each participant (treatment arm) and site visits (control arm). These data are being collected through IRIS.

Health care resource use will be detailed for a preliminary cost-consequence analysis [46,47]. The following are being reviewed: (1) software development and maintenance costs, (2) training and personnel costs for the treatment arm, (3) health care utilization data (eg, visits to the emergency department, hospital admission), and (4) other costs reported by adolescent (ie, time off from work/school). Adolescents will be asked at the post-intervention assessment to report on the use of co-interventions during the study period (eg, emergency department visits, other treatments, medication) and reasons for this use (eg, unmet need, medication prescribed before the study).

### Sample Size

We have been conservative in estimating our ability to enroll and retain participants, by assuming that 50% will refuse to participate and 50% of enrolled adolescents will drop out. A total of 160 adolescents are being invited to participate with the expectation that we will enroll 80 adolescents (40 assigned to each study arm) and retain 40 adolescents (20 per arm) by module 8 completion (post-intervention). In keeping with the standard goals of a pilot RCT, a sample size of 40 adolescents at post-intervention (20 per arm) will provide sufficient data on the primary outcome to calculate a sample size based on 90% power for a full-scale RCT [48,49].

### Treatment Condition

#### Essential Clinical Components

**Figure 2:**
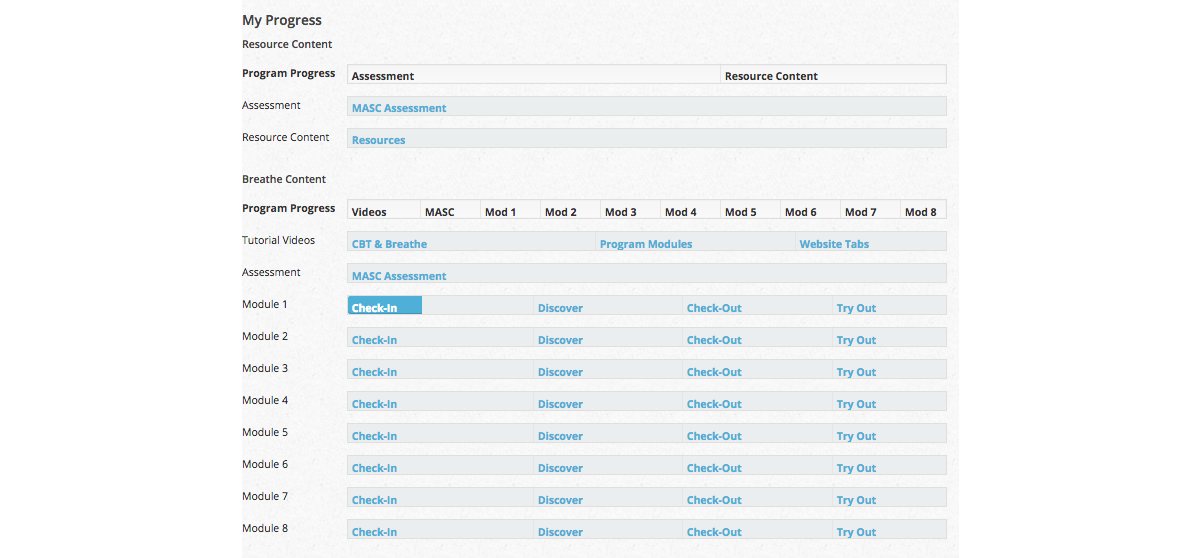
A screenshot of the Breathe program home screen.

**Figure 3:**
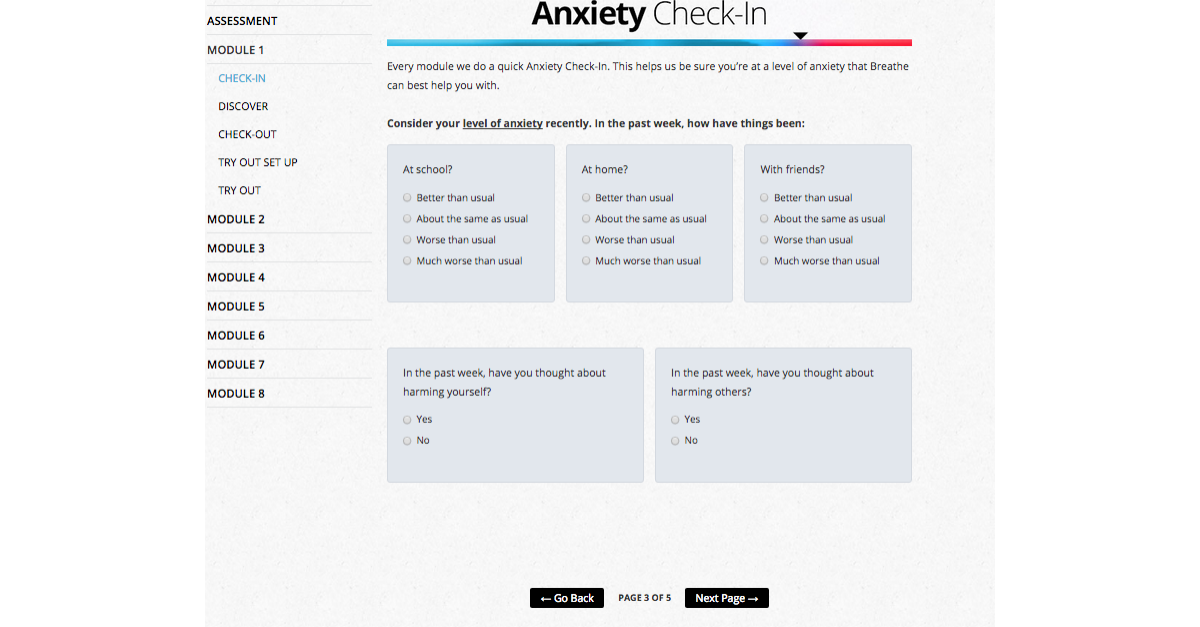
A screenshot of a weekly self-assessment activity.

**Figure 4:**
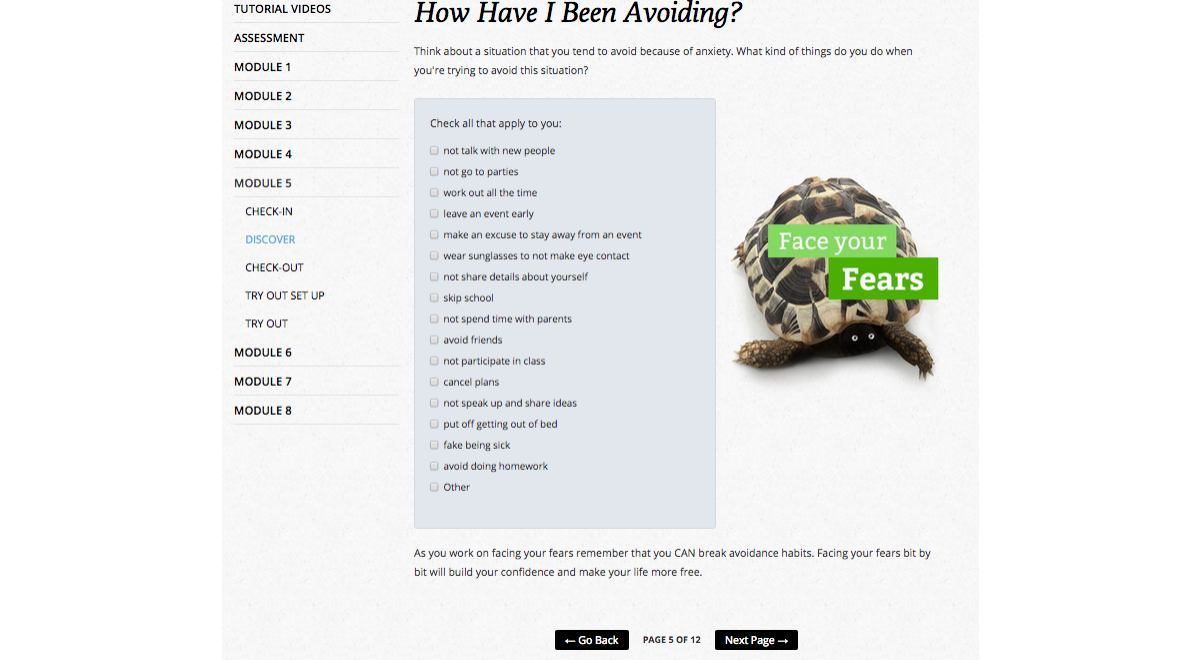
A screenshot of a learning activity.

Consistent with published CBT treatment recommendations [20,21,22], the *Breathe* program consists of 8 core CBT treatment modules (8 weeks of treatment; Figure 2) that involve (1) multi-media based education about anxiety problems and approaches to overcoming anxiety (eg, reviewing why exposure exercises are important), (2) self-assessment activities to determine level of treatment and safety needs (Figure 3), (3) activities to teach adolescents about anxiety sensitivity, how to identify anxious thoughts, and how to develop realistic thinking about anxiety-producing situations (Figure 4), (4) coping and relaxation skill activities with self-assessment of performance and rewards, (5) development of a hierarchy of feared situations and steps for gradual and repeated exposure to feared situations (using imagery and *in vivo* activities), (6) contingency management (examining the function of anxiety from a reinforcement perspective) and modeling (viewing videos of others confronting feared situations), and (7) skills for maintenance and relapse prevention. Animations, embedded video, audio playback, graphic novel style vignettes, image maps, timed prompts and on screen pop-ups provide an interactive and multimodal experience to promote sustained engagement with the treatment content across modules.

Adolescents are instructed at the beginning of the trial to use the program weekly. Adolescents who do not log in for one week receive an e-mail encouraging them to log in to complete their weekly module. During the program, adolescents have access to an *Ask the Expert* feature where they can e-mail a trained research team member with questions regarding the program and/or treatment. *Breathe* successfully underwent iterative usability testing cycles with five clinicians and five adolescents prior to the pilot study to ensure adherence to CBT therapeutic principles, developmental appropriateness for the target age group, satisfaction, and engagement with the multi-media content.

#### Persuasive Design Mechanisms

*Breathe* is delivered via the Internet-based platform IRIS, which supports the integration of persuasive design mechanisms [50] for Internet-based interventions that are proven to encourage user engagement. Through the adaptive informatics architecture of IRIS, *Breathe* personalizes the program for adolescents via three primary mechanisms: *tailoring* content (ie, providing custom information and feedback to the adolescent based on their actions); *self-monitoring *(ie, enabling the adolescent to track their own behavior toward intended outcomes); and automated *suggestions/reminders* (ie, providing adolescents with information at the right time and the right context to help them keep on track). For example, an adolescent who indicates issues with alcohol or other drug use would receive material on these topics, while an adolescent who indicates alcohol or other drug use is not an issue would not. *Breathe* also engages the adolescent’s parent(s) by sending e-mails with educational materials about the nature of adolescent anxiety and highlights of key topics their child is working on in the program. Adolescents are given the choice as to whether they want parents to receive these e-mail updates.

#### Safety Monitoring

The risks for adolescents participating in this pilot trial are considered minimal, but those in the treatment group are monitored each time they start and finish a module in the intervention program, to assess clinical functioning. Adolescents complete a *check-in* and *check-out* whereby they answer questions related to anxiety symptoms and indicate whether they are having thoughts of self-harm or harming others. This *check-in/check-out* is a typical clinical feature of CBT programs. The research team monitors the adolescents’ answers through automated indicators built into the IRIS program. If IRIS flags a safety issue (eg, decompensation in anxiety symptoms, thoughts of self-harm), a research team member contacts the adolescent and their parent(s) by phone follow-up within 36 hours and directs them to emergency services if the adolescent requires more immediate care. Additionally, the adolescent is automatically directed onscreen to a safety video on self-harm and a pop-up box appears in the program encouraging the adolescent to notify a parent/guardian of their thoughts and to seek immediate help. Access to the *Ask the Expert* feature in *Breathe* and e-mail contact also allow adolescents to identify distressing issues that may be activated during treatment. Such adolescents are re-assessed to determine if continuing in the study is advisable. Those adolescents not able to use the program independently or who are struggling with additional mental health issues may be referred to a higher level of care. Any serious adverse event will be reported to the institutional ethics board according to the standard operating procedures. Adolescents randomized to the control group are provided with contact information for local emergency resources (crisis lines, emergency department and/or other crisis mental health resources).

### Control (Minimal Intervention) Condition

Adolescents assigned to the control group are provided with access to a static webpage that houses suggested anxiety-related trade publications, print-based workbooks for adolescents, and the names of organizations and websites where the adolescent might find support. There is no interactivity or personalization included in the webpage. Adolescents assigned to the control group are provided with the option to use the *Breathe* program at the end of their 8-week control group participation. At that time, the study will follow the same processes to monitor safety and support adolescents who may require additional help, as detailed above.

### Statistical Analyses

Adolescent reports of anxiety, intervention acceptability and adherence, use of co-interventions, recruitment and retention rates, and demography will be described by numerical summaries (mean, frequency, range, and standard deviation [SD]). The mean difference in primary outcome change scores (and SDs) from baseline to the completion of module 8 (post-intervention) will be calculated with 95% CIs. SD values from the primary outcome change scores will be used to guide the full-scale RCT sample size calculations [51]. Adolescent global ratings of change (within the ranges of +2 to +3 or -2 to -3 for reported change using the 10-point Likert scale) will serve as the estimate for the MCID value [52]. The study recruitment rate (number of adolescents enrolled during the study recruitment period) and the retention rate (number of adolescents retained at the 3-month follow-up as indicated by MASC2 completion) will be calculated. These rates will be used to determine the number of study sites and time period needed for a full-scale RCT. Frequencies and proportions (with 95% CIs) will be used to describe categorical variables (eg, gender). We will summarize characteristics of completers (>75% of modules) and non-completers, and conduct tests of association to identify particular adolescents (eg, by gender) that may benefit from additional measures to maintain engagement, and will explore potential confounders (eg, age, sex, co-morbidity) that may be adjusted for in the full-scale RCT. Mean costs (and SDs) will be calculated (with 95% CIs) using published recommendations [46,47]. To account for the amortization of intervention use after upfront costs, we will distribute the investment costs over a 3- to 5-year investment time duration using a 5% societal interest rate and using 3% and 0% rates in the sensitivity analysis. Costs for reported co-interventions (health care services) will be estimated using Alberta costing and using 2015-dollar values.

## Results

Currently, adolescents are being enrolled in the study. Enrolment is taking place between March 2014 and February 2016; data collection will conclude May 2016. We expect that analysis and results will be available by August 2016.

## Discussion

As current systems-based models of health care look to enhance local community resource capacity, stepped care options and improved access to mental health services for adolescents are needed. Recent systematic reviews have shown that self-led, computer-based and computer-assisted CBT are viable treatment approaches for adolescents, but suggest that maturation of the evidence base is needed [30,31,32]. This pilot RCT is an essential step to designing a robust RCT to evaluate the effectiveness of Internet-based CBT for adolescents with moderate to mild anxiety problems. As a first step, satisfaction and treatment adherence results will indicate whether we need to make any critical intervention changes prior to a full-scale RCT.

We will use our pilot RCT results to develop a protocol for a full-scale trial. This trial will be designed to test the effectiveness of the *Breathe* program compared to a web-based, minimal control intervention providing psychoeducation (static webpage) in reducing anxiety symptoms in adolescents with moderate to mild anxiety problems. The MCID, sample calculation, and co-intervention use determined from the pilot RCT will inform full-scale trial methodology. Our sample calculation will ensure the full-scale trial is adequately powered, and will address sample size limitations noted in the literature to date [30]. The use of the MCID in the full-scale trial may also help to clarify inconclusive self-rated treatment effects reported across similar trials [30]. Our cost-consequence analysis will inform a cost-effectiveness analysis in the full-scale RCT. Finally, we will use the recruitment and retention rates calculated in the pilot trial to determine the number of study sites and timeline necessary to recruit the full-scale RCT sample.

## Abbreviations

ASQ: Ask Suicide-Screening Questions
Breathe: Being Real, Easing Anxiety: Tools Helping Electronically
CBT: cognitive behavioral therapy
IRIS: Intelligent Research Intervention Software
MASC: Multidimensional Anxiety Scale for Children
MCID: minimal clinically important difference
RCT: randomized controlled trial
SCARED: Screen for Child Anxiety Related Emotional Disorders
SD: standard deviation

## Multimedia Appendix 1

Peer reviewer comments from the CIHR grant supporting this study. This manuscript reports Phase 3 (the pilot RCT) that is commented on in the reviews.
