# Supplementary material for: Increasing Access to Mental Health Care With Breathe, an Internet-Based Program for Anxious Adolescents: Study Protocol for a Pilot Randomized Controlled Trial
Source: JMIR Res Protoc. 2016 Jan 29;5(1):e18. doi: 10.2196/resprot.4428 (PMC4752691; doi:10.2196/resprot.4428)
Supplement: Supplementary file 1 [file resprot_v5i1e18_app1.pdf]

Review Type/Type d'évaluation: Committee Member 1/Membre de comité 1
Name of Applicant/Nom du chercheur: NEWTON, Mandi Shantell
Application No./Numéro de demande: 258892
Agency/Agence: CIHR/IRSC
Competition/Concours: 2011-09-15 Operating Grant/Subvention de fonctionnement
Committee/Comité: Social & Developmental Aspects of Children's & Youth's Health/Aspects sociaux et développementaux de la santé de l'enfant et de l'adolescent
Title/Titre: Developing an Internet-based program for anxious youth who visit the emergency department for mental health care: A strategy to increase access to post-crisis care

---

Assessment/Évaluation:

"Developing an Internet-based program for anxious youth who visit the emergency department for mental health care: A strategy to increase access to post-crisis care"

Newton, Mandi Shantell (Nominated PA); McGrath, Patrick John (PA); Baxter (Co-A); Carandang (Co-A); Curtis (Co-A); Fitzpatrick (Co-A); Jabbour (Co-A); Johnson (Co-A); Joyce (Co-A); Ohinmaa (Co-A); Rosychuk (Co-A); & Young (Co-A)

SYNOPSIS OF PROPOSAL
The overall purpose of the proposed project is to develop and carry out preliminary testing of an Internet-based cognitive behavioural therapy (CBT) for anxious youth (13-17 years) having visited an emergency department (ED). It is a 3 year project entailing 3 studies:

1. Study 1: Internet-based intervention development (goal 1)
2. Study 2: Real-time intervention evaluation of usability (goal 2)
3. Study 3: Preliminary data for a future multi-site RCT (goal 3)

Applicants have provided their response to reviewers regarding their earlier submission.

RESEARCH APPROACH
Through the project, *Wired!*, an Internet-based CBT program for youth will be developed. Each of the three studies has specific objectives, design, and methods.

<u>In Study 1</u>, 8 CBT modules will be developed for *Wired!* which will include process (e.g. assessment of module upon completion), core content (e.g. coping skill activities, relapse prevention), and tailored (e.g. mood/behaviour, assertiveness training) modules. The approach follows Dr. McGrath's *Feeling Better* program development. A Youth Panel (n=10) and a Clinical Expert Panel (n=10) will be formed through maximum variation sampling and convened through *Elluminate Live* (online interaction). Through either focus groups or individual semi-structured interviews, panel members will provide input on module content. *For the youth panel, youth 17-24 years will be recruited. However, the focus of the intervention is on youth 13-17 years. What is the rationale for the age variation?* Data will be entered into N-Vivo. *Is use of a computer assisted qualitative data analysis necessary given the sample sizes above (10+ 10) and specific objectives of this phase of the study?*

Review Type/Type d'évaluation: Committee Member 1/Membre de comité 1
Name of Applicant/Nom du chercheur: NEWTON, Mandi Shantell
Application No./Numéro de demande: 258892
Agency/Agence: CIHR/IRSC
Competition/Concours: 2011-09-15 Operating Grant/Subvention de fonctionnement
Committee/Comité: Social & Developmental Aspects of Children's & Youth's Health/Aspects sociaux et développementaux de la santé de l'enfant et de l'adolescent
Title/Titre: Developing an Internet-based program for anxious youth who visit the emergency department for mental health care: A strategy to increase access to post-crisis care

Assessment/Évaluation:

In Study 2, the usability (efficiency, errors, and acceptability) of *Wired!* will be evaluated with participants from the Youth Panel (n=8 to 10) with assistance from a usability analyst. The evaluation process is innovative (building on previous work conducted by Dr. McGrath) and iterative (2-3 cycles or until no further problems are identified).

Study 3, entails a demonstration RCT with an Internet-delivered CBT through *Wired!* (experimental, n=40) group and a resource webpage (control, n=40) group (with retention goal of 20 per group at 3 month follow-up). The goal is to evaluate methodological processes and outcomes (e.g. sample size estimation, changes in anxiety levels pre and post-intervention) in preparation for a full-scale RCT in the future by gathering relevant information at this phase. Participants will be recruited from an Edmonton and a Halifax health care site.

Among the strengths of the proposal are its originality and potential impact (see below sections). The project builds on previous work conducted in the field and will contribute to new knowledge development. Also two study potential challenges are considered (1. Trial may affect usual ED treatment, and 2. Risk management concerns in treatment trials for youth). Appendix 1 outlines information on the proposed research staff training workshop.

As per information presented in the proposal's background section, anxiety disorders (AD) are among the most prevalent mental health problems in children and youth. This section also notes that AD rates are comparable among boys and girls. *It would be helpful to elaborate how the authors came to this conclusion given other bodies of literature pointing to gender differences, with females being at higher risk of AD.* Otherwise, the proposal has a comprehensive and well-written review of literature of AD in youth and challenges to use of computer-assisted treatments

Given the multi-study aspect of the proposed project and specific outcomes expected for each of the 3 studies, a potential threat to it may be its timely completion in three years. *Does the research team have contingency plans in case of unexpected delays for each of the studies*?

ORIGINALITY OF PROPOSAL
Delivering CBT to youth with AD is a new area of research and practice. However, the proposal draws from work in related fields and individualizes it to youth with AD. To this end, the multi-stage innovative project

**Review Type/Type d'évaluation:**	Committee Member 1/Membre de comité 1
**Name of Applicant/Nom du chercheur:**	NEWTON, Mandi Shantell
**Application No./Numéro de demande:**	258892
**Agency/Agence:**	CIHR/IRSC
**Competition/Concours:**	2011-09-15 Operating Grant/Subvention de fonctionnement
**Committee/Comité:**	Social & Developmental Aspects of Children's & Youth's Health/Aspects sociaux et développementaux de la santé de l'enfant et de l'adolescent
**Title/Titre:**	Developing an Internet-based program for anxious youth who visit the emergency department for mental health care: A strategy to increase access to post-crisis care

Assessment/Évaluation:

will combine clinical knowledge with technological tools to deliver CBT for youth AD. It has the potential to inform delivery of better follow-up and continuous care for youth who may otherwise not have access to ongoing mental health services.

APPLICANTS

In combination the team have content and methodological expertise to carry out the project. The team is multidisciplinary (medicine, nursing, psychology, public health) and multi-site (University of Alberta, IWK Health Centre – Halifax, University of Calgary, McMaster University), with expertise in child psychiatry and psychology and emergency care. Dr. Newton (Nominated PA) has expertise in CBT and computer-based intervention evaluation. Dr. Patrick McGrath (PA) is developer of the Strongest Families Program and has conducted large scale studies with parents and children. Among the strengths of the proposal is identification of specific study roles of each team member and collaborator (in Appendix F).

ENVIRONMENT FOR RESEARCH

Content development for *Wired!* will take place in Edmonton (led by Dr. Newton) and web development will take place in Halifax (led by Dr. McGrath). Recruitment of youth will take place in Edmonton and Halifax. Team members have prior and developed connections with agencies and professionals both in the clinical and technological aspects of the proposed project.

IMPACT OF RESEARCH

Given the potential high appeal of the on-line medium for most youth, lack of comprehensive follow-up for youth with AD following their ED visit, and potential for improvement in outcome through CBT, the project can be highly relevant to youth (and their families) with AD.

BUDGET

Total Requested (1st year): $154,774 (with $3,450 in-kind support)

Total Funds Requested: $412,582 (3 years of support requested)

Personnel costs include salary: Two 0.5 FTE Research Coordinators (Years 1-3) (1 at Edmonton Site and 1 at Halifax site),

**Review Type/Type d'évaluation:**	Committee Member 1/Membre de comité 1
**Name of Applicant/Nom du chercheur:**	NEWTON, Mandi Shantell
**Application No./Numéro de demande:**	258892
**Agency/Agence:**	CIHR/IRSC
**Competition/Concours:**	2011-09-15 Operating Grant/Subvention de fonctionnement
**Committee/Comité:**	Social & Developmental Aspects of Children's & Youth's Health/Aspects sociaux et développementaux de la santé de l'enfant et de l'adolescent
**Title/Titre:**	Developing an Internet-based program for anxious youth who visit the emergency department for mental health care: A strategy to increase access to post-crisis care

Assessment/Évaluation:

Research Trainees: 1 Graduate Student (Year 1-3, under Dr. Newton's supervision), 1 Postdoctoral (Years 2-3 under Dr. McGrath's supervision)

Review Type/Type d'évaluation: Committee Member 2/Membre de comité 2
Name of Applicant/Nom du chercheur: NEWTON, Mandi Shantell
Application No./Numéro de demande: 258892
Agency/Agence: CIHR/IRSC
Competition/Concours: 2011-09-15 Operating Grant/Subvention de fonctionnement
Committee/Comité: Social & Developmental Aspects of Children's & Youth's Health/Aspects sociaux et développementaux de la santé de l'enfant et de l'adolescent
Title/Titre: Developing an Internet-based program for anxious youth who visit the emergency department for mental health care: A strategy to increase access to post-crisis care

Assessment/Évaluation:

This is a resubmission of a previous application. The rationale for this proposal is that anxiety disorders are quite prevalent in youth and are a common cause for emergency room visits. However, due to lack of resources in the community, there is delayed time for post crisis treatment with a median length of 35 days. Internet based delivery of cognitive behavior therapy for anxiety may help to reduce anxiety related crisis and reduce repeat emergency room visits.

This is a 3-phase application, with year one focusing on development, refinement and pre-testing of a web-based intervention. The second part of the research plan will involve a qualitative study with stakeholders helping to refine the content and delivery features. This will then be pre-tested by youth. Lastly, a pilot randomized control trial will form the basis for the remaining aspect of the study. Youth ages 13 to 17 will be recruited following discharge from the emergency department after presenting for anxiety. They will be randomized to either the internet CBT group or a control group, which is a website providing general information on resources for anxiety. Pre and post measures will include change in youth anxiety from a validated measure (MASC) from baseline to 3 months. The length of the intervention is weeks. Data from this will also be used to estimate a sample recruitment and retention rate for future large scale RCT to determine important differences in anxiety change measure intervention acceptability and determine the use of co-interventions.

This application has numerous strengths. The applicants have revised their application to address the previous reviewer’s comments in a very detailed and thoughtful manner. This has significantly strengthened their current application. The significance of the issue is significant for Canadian youth and this study will fill a gap in this area for this particular age group who have not yet been studied using similar methodologies. The applicants have thought through many of the details extensively as evidenced by the appendices with the grant. Other positive inclusions are the use of CBT as a treatment method and the ability of the program to be tailored to the individual youth, along with safety measures that have been included for the subjects.

The investigators seem well established and have the necessary expertise to carry out this study. The investigators modestly assume that 50% of potential subjects will decline to enter, however given the acute situation and the interest in receiving care, one may actually assume that a higher participation rate will ultimately occur. Statistical methods appear appropriate.

It was actually very difficult to find the duration of the intervention in the grant application as it is not stated

**Review Type/Type d'évaluation:**	Committee Member 2/Membre de comité 2
**Name of Applicant/Nom du chercheur:**	NEWTON, Mandi Shantell
**Application No./Numéro de demande:**	258892
**Agency/Agence:**	CIHR/IRSC
**Competition/Concours:**	2011-09-15 Operating Grant/Subvention de fonctionnement
**Committee/Comité:**	Social & Developmental Aspects of Children's & Youth's Health/Aspects sociaux et développementaux de la santé de l'enfant et de l'adolescent
**Title/Titre:**	Developing an Internet-based program for anxious youth who visit the emergency department for mental health care: A strategy to increase access to post-crisis care

Assessment/Évaluation:

in the methods (8 weeks was seen in one of the appendices). Given the median time is 35 days to receive treatment, one would assume that the majority of the participants will be receiving other additional therapy outside of the study design. The authors do not address this nor how this co-intervention will be accounted for. Secondly, the background does highlight that many of these youth may have repeat visits to the emergency room prior to receiving care in the community. It would be helpful to know the actual statistics in regards to this and whether this outcome will also be captured in this study.

Budget seems adequate for the scope of the study.

The principal applicant is a relatively new investigator with an interest in emergency utilization and mental health. She has published 38 manuscripts, almost half within the last 2 years. She holds a CIHR New Investigator Salary Award Research award and a CIHR Canadian Child Health Clinician Scientist Program Career Development Award. She is well supported by the co-PI who is a senior investigator with experience in clinical trials and recent research activity in the mental health field.
